# Multi-Compartment Transcriptomics Identifies a Persistent Inflammatory Program and a Network-Derived Diagnostic Signature in Polycythemia Vera

**DOI:** 10.3390/ijms27104580

**Published:** 2026-05-20

**Authors:** Abdulmohsen M. Alruwetei

**Affiliations:** Department of Medical Laboratories, College of Applied Medical Sciences, Qassim University, Buraydah 51431, Saudi Arabia; roietaie@qu.edu.sa

**Keywords:** polycythemia vera, JAK2V617F, transcriptomics, gene expression, hematopoietic stem/progenitor cells, neutrophils, inflammatory response, JAK–STAT, GSEA

## Abstract

Polycythemia vera (PV) is a JAK2V617F-driven myeloproliferative neoplasm characterized by erythroid expansion, increased thrombotic risk, and heterogeneous clinical outcomes. Although prior studies have described key transcriptional abnormalities—including Janus kinase–signal transducer and activator of transcription (JAK–STAT) hyperactivation and chronic myeloinflammation—most have examined single hematopoietic compartments. A multi-compartment approach may reveal conserved and lineage-specific disease-associated transcriptional programs. Here, an integrated, multi-compartment transcriptomic analysis of publicly available microarray datasets was performed, spanning bone marrow (BM) CD34+ progenitors, peripheral blood (PB) CD34+ progenitors, and whole blood from PV patients and healthy controls, with independent validation in neutrophils. Differential gene expression, pathway enrichment, and protein–protein interaction network analyses were used to delineate conserved versus compartment-specific transcriptional programs and to evaluate persistence of progenitor-derived signatures into mature myeloid cells. Across compartments, PV demonstrated consistent enrichment of inflammatory, interferon, and JAK–STAT-associated pathways despite limited overlap at the individual gene level, indicating that core disease processes are maintained through lineage- and differentiation-stage-specific transcriptional reprogramming. Network analysis identified highly connected hub genes, which were used to derive a single-sample gene set enrichment (ssGSEA) signature. This signature showed strong diagnostic performance across cohorts; remained enriched in PV neutrophils; and correlated with platelet count, indolent disease status, and reduced levels in post-splenectomy patients. Together, these findings support a model in which PV is driven by stable, progenitor-derived inflammatory programs that persist across myeloid differentiation while incorporating compartment-specific adaptations, and highlight the value of multi-compartment, network-based approaches for translational biomarker development.

## 1. Introduction

The polycythemia vera (PV) is a chronic clonal myeloproliferative neoplasm (MPN) characterized by erythrocytosis and driven in most patients by the JAK2V617F mutation, which leads to constitutive JAK–STAT pathway hyperactivity in myeloid lineage cells and is often accompanied by leukocytosis and thrombocytosis [1,2,3,4]. This pathogenic process also drives a chronic pro-inflammatory state characterized by excessive production of cytokines, endothelial dysfunction, enhanced platelet–leukocyte interactions, and increased neutrophil extracellular trap formation (NETosis), all of which contribute to the high thrombotic risk and broad-symptom burden observed in patients with PV [3,5,6,7,8]. Management of PV focuses primarily on reducing risk of thrombosis by suppressing clonal proliferation when indicated, using cytoreductive drugs to alleviate increased hematocrit, leukocyte, and platelet counts. These measures help limit blood hyperviscosity and attenuate inflammatory activation [1,3,9].

Prior transcriptomic studies have consistently revealed substantial gene and pathway-level alterations relevant to PV pathogenesis, including JAK–STAT signaling genes and myeloid-inflammatory signatures [2,10,11,12,13]. However, most studies have assessed single compartments: whole blood, peripheral blood (PB) CD34+ cells, or bone marrow (BM) CD34+ cells, each providing only a partial view of the transcriptional activities that represent the disease biology [11,14]. A multi-compartment transcriptomic framework enables identification of transcriptional programs conserved across hematopoietic differentiation, distinguishing core, lineage-independent pathogenic mechanisms from differentiation-dependent responses, while capturing lineage-specific modulation with biological and clinical relevance [12,15,16].

This study carried out an integrated transcriptomic analysis across multiple hematopoietic compartments in PV. Publicly available microarray datasets were harmonized to compare BM CD34+ cells, PB CD34+ cells, and whole blood, with validation performed in an independent neutrophil cohort. The selected compartments represent complementary stages of hematopoietic differentiation relevant to PV biology. Bone marrow CD34+ cells capture early progenitor-level transcriptional programs where the JAK2-mutant clone originates. PB CD34+ cells represent circulating progenitors and enable assessment of disease propagation beyond the marrow niche. Whole blood reflects the integrated transcriptional output of mature circulating cells, providing a system-level view of disease manifestation. Neutrophils, as terminally differentiated myeloid effector cells central to PV-associated inflammation and thrombosis, were included as an independent validation cohort to test whether progenitor-derived disease programs persist into clinically relevant mature lineages.

Accordingly, this study aimed to (1) define conserved and compartment-specific transcriptional signatures; (2) map the core dysregulated pathways and their associated protein-interaction network; and (3) evaluate the clinical utility of a diagnostic signature derived from network hub genes.

## 2. Results

### 2.1. Differential Gene Expression Analysis Across Hematopoietic Compartments

**Figure 1:**
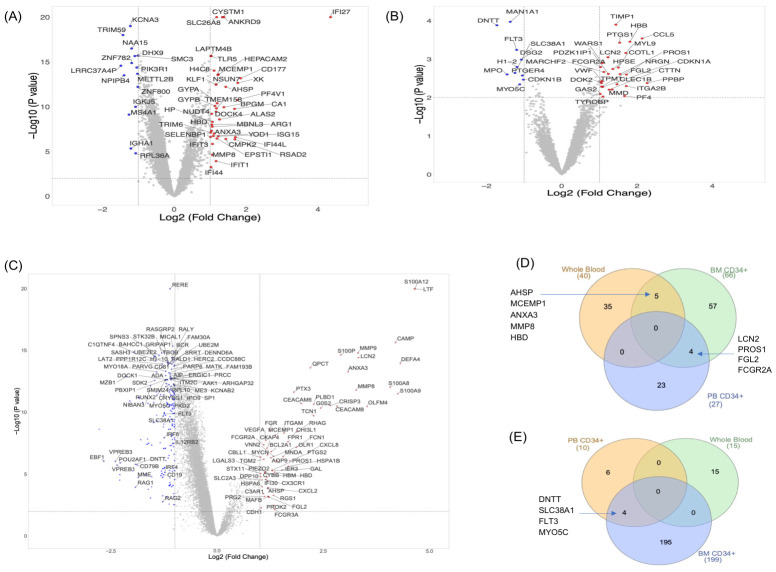
Differential gene-expression analysis comparing PV patients with HC across multiple hematopoietic compartments. (**A**–**C**). Volcano plots for whole blood, PB CD34+ cells, and BM CD34+ cells, respectively. For (**C**) BM CD34+ cells, gene labels denote all significantly upregulated genes, the top 50 significantly downregulated genes, and shared significantly downregulated genes across hematopoietic compartments. Venn diagram analysis showing the overlap of DEGs across datasets in (**D**) upregulated and (**E**) downregulated genes. Red indicates significantly upregulated (overexpressed) genes, while blue indicates significantly downregulated genes based on the defined thresholds. The dotted lines represent thresholds of log_2_ fold change (±1) and −log_10_ (*p*-value) > 2.

The significant change in gene expression between PV and healthy controls (HCs) in each cohort was determined using thresholds of log_2_ fold change >±1 and –log_10_(*p*-value) > 2, as shown in the volcano plots in Figure 1A–C. In whole blood, 40 upregulated genes were detected; the upregulated signature was dominated by neutrophil/innate immune-associated genes (e.g., *CD177*, *ARG1*, *TLR5*, *LAPTM4B*, and *CYSTM1*), erythroid markers (e.g., *AHSP*, *KLF1*, *BPGM*, *GYPA*, *ALAS2*, *CA1*, and *XK*), and interferon-stimulated genes (e.g., *IFI44L*, *RSAD2*, *IFIT1/3*, *IFI27* and *ISG15*). Fifteen genes were downregulated, including Lymphoid/B-cell/T-cell associated genes (e.g., *IGKJ5*, *IGHA1*, *MS4A1*, *KCNA3*, and *PIK3R1*) (Figure 1A). The PB CD34+ cohort showed 27 upregulated and 10 downregulated genes, with upregulation enriched in myeloid/megakaryocytic differentiation markers (e.g., *HBB*, *ITGA2B*, *VWF*, *MYL9*, *TPM1*, *CLEC1B*, and *NRGN*) and inducible inflammatory markers (e.g., *CCL5*, *LCN2 CDKN1A*, *FGL2*, *WARS1*, and *COTL1*), and downregulation of a mixed set, including early/lineage-commitment genes (e.g., *DNTT*, *MAN1A1*, *FLT3*, *DSG2*, *MPO*, *H1-2*, *SLC38A1*, *PTGER4*, *CDKN1B*, and *MYO5C*) (Figure 1B).

The BM CD34+ cohort demonstrated the most prominent transcriptional dysregulation (66 upregulated; 199 downregulated) in this analysis. The overexpressed genes included alarmins (*S100A8/A9/A12*), neutrophil granule proteins and effectors (*DEFA4*, *CAMP*, and *OLFM4*), cytokines, chemokines and inflammatory mediators (*CXCL8*, *CXCL2*, *VEGFA*, *PTGS2*, *LCN2*, and *FGL2*), and myeloid cell surface receptors and adhesion molecules (*ITGAM*, *FCGR3A*, *FCGR2A*, *FPR1*, and *FGR*). Marked downregulation was detected in genes involved in early and immature B-cell development (e.g., *DNTT*, *RAG1*, *RAG2*, *VPREB1*, *VPREB3*, *CD19*, *CD79B*, *EBF1*, *POU2AF1*, *IRF4*, *IRF8*, *FLT3*, *IL12RB2*, and *MME*) (Figure 1C).

**Figure 2:**
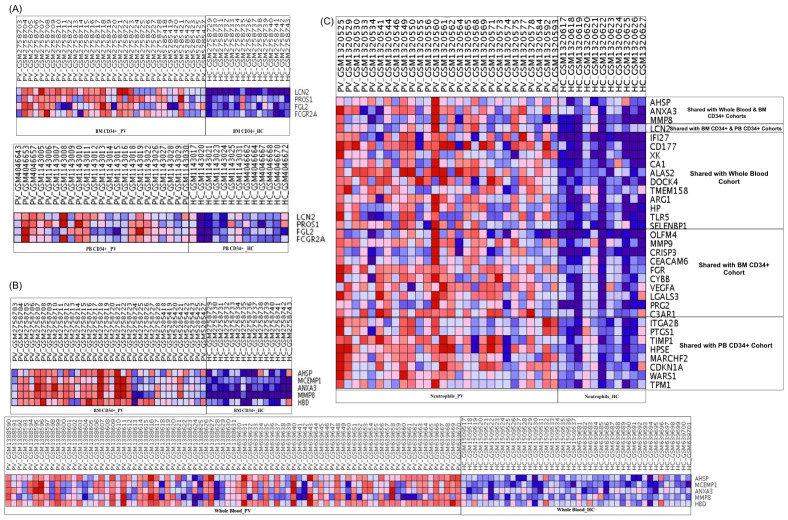
Heatmap presentation of shared overexpressed genes in PV between cohorts. Heatmaps in (**A**–**C**) visualize shared upregulated genes: (**A**) between BM and PB CD34+ cohorts, (**B**) between BM CD34+ and whole blood, and (**C**) across all study cohorts plus the neutrophil validation cohort. Expression levels are shown in a blue-to-red gradient, where blue indicates downregulation and red indicates overexpression.

Cross-cohort analysis of DEGs was performed using a Venn diagram to identify transcriptional signatures in PV that were consistently shared across early and mature myeloid lineage cells, revealing both compartment-specific and overlapping gene expression patterns. Among the upregulated genes, none was common to all three compartments; however, pairwise intersections uncovered conserved signatures. A core inflammatory signature (*LCN2*, *PROS1*, *FGL2*, and *FCGR2A*) was conserved between BM CD34+ and PB CD34+ progenitors (Figure 1D and Figure 2A). Whole blood and BM CD34+ cells shared five genes (*AHSP*, *ANXA3*, *HBD*, *MMP8*, and *MCEMP1*), reflecting erythroid and myeloid/inflammatory overlap (Figure 1D and Figure 2B). Overlap analysis of the downregulated genes revealed four common genes (*DNTT*, *SLC38A1*, *FLT3*, and *MYO5C*) that were consistently reduced in PV of BM CD34+ and PB CD34+ cohorts (Figure 1E).

To evaluate persistence of the identified signatures and independently validate the results, upregulated genes from the cohorts were examined in an external neutrophil validation cohort. The analysis confirmed sustained differential overexpression in PV neutrophils relative to HCs, including genes commonly overexpressed in whole blood and BM CD34+ cohorts (e.g., *AHSP*, *ANXA3*, and *MMP9*), as well as one gene, *LCN2*, which was overexpressed in both PB CD34+ and BM CD34+ cohorts (Figure 2C). Furthermore, the concordant overexpression pattern in neutrophils of PV was also observed for a range of genes restricted to individual compartment (e.g., *IFI27* and *CD177*) (Figure 2C). This finding suggests that PV is characterized by a continuous transcriptional program extending from myeloid progenitors to mature myeloid effector cells.

### 2.2. Hallmark Gene Set Enrichment Analysis (GSEA)

**Figure 3:**
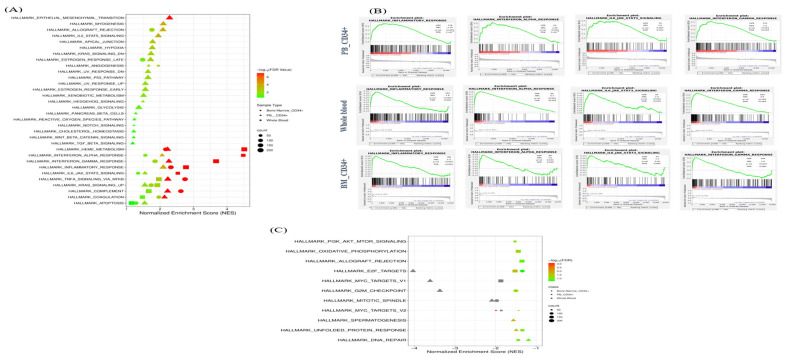
Gene Set Enrichment Analysis (GSEA) of Hallmark pathways in PV versus HCs. Summary of positively and negatively enriched Hallmark pathways across whole blood, PB CD34+, and BM CD34+ datasets: (**A**) Bubble plot showing significantly upregulated Hallmark pathways, illustrating pathway magnitude and cohort-specific patterns. (**B**) Representative GSEA enrichment curves for selected upregulated pathways. Shown are the normalized enrichment score (NES), gene set size (SIZE), nominal *p*-value (*p*-val), and FDR for each pathway. (**C**) Bubble plot of significantly downregulated Hallmark pathways. Grey-colored points indicate pathways with −log_10_(FDR) values exceeding the display threshold and not represented within the color scale; values were capped at 30.

To investigate how key biological processes differed between PV and HC across all study cohorts, GSEA was performed using the Hallmark gene sets database. Significantly enriched biological pathways were determined based on an FDR < 0.25 (Figure 3A–C). Positively enriched pathways—those upregulated in PV—were prominent across all compartments, with 32, 13, and 10 significant gene sets identified in the PB CD34+, BM CD34+, and whole blood cohorts, respectively (Figure 3A). Ten Hallmark gene sets were commonly enriched across the study cohorts, including the Inflammatory Response, Interferon Alpha Response, Interferon Gamma Response, and IL-6/JAK–STAT3 Signaling (Figure 3A,B). In contrast, two negatively enriched Hallmark gene sets that were common across cohorts were identified—MYC targets and E2F targets. Additional negatively enriched pathways included G2M Checkpoint, Mitotic Spindle, Oxidative Phosphorylation, and DNA Repair (Figure 3C). The reduced activity of these pathways in PV suggests a relative suppression of proliferative transcriptional programs. Collectively, the findings emphasize strong transcriptional dysregulation of innate immune and inflammatory processes in PV across hematopoietic compartments, concurrent with attenuated proliferative signals from E2F- and MYC-driven signals, potentially reflecting the chronic signaling-induced feedback and exhaustion.

### 2.3. Protein–Protein Interaction (PPI) Network Analysis

**Figure 4:**
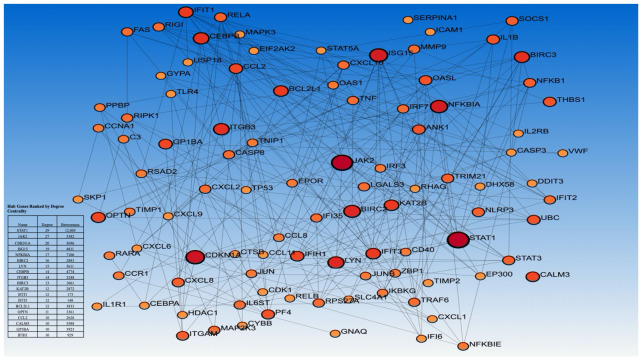
Protein–protein interaction (PPI) network of common leading-edge genes from positively enriched pathways in PV. The network displays interactions among genes identified as shared drivers of pathway enrichment across study cohorts. Nodes represent proteins, with larger node sizes indicating higher degree centrality. Node colors range from orange to dark red, reflecting increasing degree centrality (higher connectivity). Edges (lines) represent protein–protein interactions between genes. The inset table lists the top-ranked hub gene.

The PPI analysis was conducted on STRING v12.0 using the leading-edge genes derived from the ten Hallmark gene sets commonly upregulated across the study cohorts to map the interactive associations among core overexpressed genes in PV. Topological analysis revealed 341 nodes and 698 edges with an average node degree of 4 (range: 1–29). Key hub genes were defined as nodes with degrees ≥ 10 (*n* = 18). Key hubs and their node degrees include STAT1 (29), JAK2 (27), CDKN1A (20), ISG15 (19), NFKB1 (17), BIRC2 (16), LYN (15), CEBPB and ITGB3 (14), BIRC3 (13), KAT2B, IFIT1, and IFIT3 (12), OPTN (11), and CCL2, CALM3, GP1BA, and IFIH1 (10) (Figure 4). These highly connected genes are central regulators of inflammatory, cytokine, and interferon-response pathways—most notably JAK–STAT, NF-κB, and interferon signaling—highlighting their pivotal roles in the transcriptional dysregulation observed in PV.

### 2.4. Diagnostic Performance of the Hub-Gene Signature

**Figure 5:**
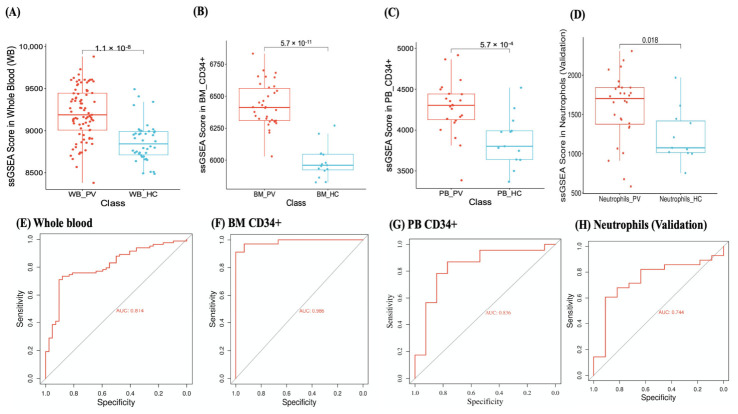
Discriminatory performance of high-degree node (hub gene) signatures in distinguishing PV from HCs. ssGSEA scores derived from hub genes were compared between PV and HCs across study cohorts and then validated in the neutrophil validation cohort. Boxplots show the distribution of ssGSEA enrichment scores for the hub gene signature in (**A**) whole blood, (**B**) BM CD34+ cells, (**C**) PB CD34+ cells, and (**D**) neutrophils (validation) cohort. Receiver operating characteristic (ROC) curves demonstrate the diagnostic performance of the signature in (**E**) whole blood, (**F**) BM CD34+, (**G**) PB CD34+, and (**H**) the validation cohort, with area under the curve (AUC) values shown.

To evaluate the potential diagnostic utility of the highly connected hub genes identified in the PPI network in differentiating PV from HCs, single-sample Gene Set Enrichment Analysis (ssGSEA) was applied to obtain an enrichment score for each sample, representing the coordinated activity of this hub-gene set. The diagnostic performance of the signature was first evaluated in the three study cohorts. ssGSEA scores were significantly elevated in PV patients compared with controls (whole blood: *p* = 1.10 × 10^−^^8^; BM CD34+: *p* = 5.7 × 10^−^^11^; PB CD34+: *p* = 5.7 × 10**^−^**^4^) (Figure 5A–C). The signature demonstrated high discriminative accuracy, with areas under the ROC curve (AUC) of 0.814 for whole blood, 0.986 for BM CD34+, and 0.836 for PB CD34+ (Figure 5E–G). The ssGSEA-based signature was subsequently validated in the neutrophil cohort, where enrichment scores remained significantly higher in PV samples (*p* = 0.018; Figure 5D) and achieved an AUC of 0.744 (Figure 5H).

### 2.5. Association of Hub-Gene ssGSEA Signature with PV Clinical Features

To assess the clinical relevance of the hub-gene signature in PV, ssGSEA scores were integrated with clinical metadata from the GSE47018 PB CD34+ cohort (*n* = 19 PV patients) [12]. The metadata included key categorical clinical variables, such as gender (8 males; 11 females), history of splenectomy (*n* = 5), prior thrombotic events (*n* = 5), leukemic transformation (*n* = 5), and disease aggressiveness (12 indolent vs. 7 aggressive cases). In addition, several clinical parameters were assessed, including patient age, JAK2V617F allele burden, disease duration, hemoglobin levels, leukocyte counts, and platelet counts. The analysis revealed significant associations with disease aggressiveness (*p* = 0.014) and splenectomy status (*p* = 0.042). Specifically, the signature was higher in patients with indolent disease and in those without a history of splenectomy. Associations with survival and acute leukemia transformation were not statistically significant (Table 1). Spearman correlation analysis further identified a significant positive correlation between the signature and platelet count (ρ = 0.049). No significant correlations were observed with other hematological parameters (Table 1).

## 3. Discussion

Polycythemia vera (PV) is characterized by well-documented transcriptional dysregulations that have advanced understanding of its pathogenesis. However, it remains essential to clarify the extent to which these transcriptional programs are persistent and tissue-specific, as this has important implications for disease biology, biomarker development, and compartment-specific therapeutic strategies [17]. Addressing this gap, this study systematically integrates microarray gene-expression data across multiple hematopoietic compartments in PV, providing new insight into the persistence of disease-associated programs across differentiation. By analyzing heterogeneous cell populations across distinct stages of hematopoietic maturation, transcriptional differences reflecting both lineage-specific differentiation programs and PV-associated disease biology could be evaluated. This intentional incorporation of cellular heterogeneity enabled discrimination between conserved disease-associated transcriptional programs that persist across differentiation and compartment-specific expression patterns characteristic of individual cell types.

The DEGs analysis identified a core set of conserved genes with inflammatory activity in PV across compartments, suggesting their role as stable molecular features of PV and that inflammatory signals established at early progenitor stages rather than arising only in mature myeloid cells. In support of this, a common inflammatory core (*LCN2*, *PROS1*, *FGL2*, and *FCGR2A*) was identified between BM CD34+ and PB CD34+. These genes are repeatedly linked to PV and other MPNs consistent with their known functions in neutrophil activation, immune dysregulation, and pro-thrombotic signaling [12,16,18,19,20,21]. Similarly, five erythroid-associated and inflammatory transcripts (*AHSP*, *ANXA3*, *HBD*, *MMP8*, and *MCEMP1*) were detected in whole blood and BM progenitors, indicating for the well-known coexistence of erythroid skewing and chronic inflammation in PV, a defining feature of the disease [22,23]. Validation in neutrophils confirmed the persistence of conserved genes (*AHSP*, *ANXA3*, *LCN2*, and *MMP8*), highlighting the strong biological consistency of this gene subset. The repeated detection of this subset is also in line with reports showing chronic inflammatory priming of JAK2V617F-mutant progenitors [24,25]. Notably, despite these conserved features, the overall limited overlap of DEGs across compartments indicates that core inflammatory and JAK–STAT-driven pathways are maintained through lineage- and differentiation-stage-specific transcriptional regulation rather than uniform gene expression across cell types. This distinction provides a conceptual explanation for the variability of gene-level signatures reported across prior transcriptomic studies in polycythemia vera, while reinforcing inflammatory and JAK–STAT signaling as stable disease drivers [12,26,27].

Compartment-specific programs also emerged in the DEGs. BM CD34+ cells showed induction of neutrophil-associated effectors (e.g., *OLFM4* and *MMP9*), while PB CD34+ cells displayed megakaryocytic and platelet-related activation (e.g., *ITGA2B* and *PTGS1*). Whole-blood-specific changes were also evident, including upregulation of interferon-responsive transcripts such as the well-recognized interferon-stimulated gene *IFI27*, along with the neutrophil activation marker *CD177*. A concordant pattern of overexpression was also observed in the independent PV neutrophil validation cohort for these genes and other compartment-restricted transcripts, reinforcing the continuity of these transcriptional programs across myeloid maturation [14,15]. Overall, these observations support a model in which JAK2-mutant myeloid cells promote thrombo-inflammatory processes through increased neutrophil activation, endothelial interaction, and NET formation—mechanisms extensively documented in MPN-related thrombosis [28,29].

Pathway enrichment analysis showed uniform activation of inflammatory, interferon-α/γ, and IL-6/JAK–STAT3 signaling across compartments, emphasizing the findings from the gene expression analysis that the disease is sustained by chronic inflammatory pathways [30,31]. Network analysis identified central regulators (STAT1, JAK2, CDKN1A, and NFKB1), highlighting their integrative roles in inflammatory signaling and clonal fitness [32,33]. These hubs are consistent with multiple genomic and transcriptomic studies repeatedly reporting dysregulation of JAK–STAT, NF-κB, and interferon pathways in PV and underscore the potential value of targeting inflammatory pathways to reduce disease complications [24]. The creation of an ssGSEA score based on the hub-gene signature demonstrated strong diagnostic performance across all cohorts, and the consistency of this signature in the independent cohort and biological contexts supports the relevance of the identified transcriptional program to core disease processes in PV and its exploratory diagnostic value, although validation in larger, well-annotated datasets is still needed.

The hub-gene ssGSEA score was also associated with platelet count, indolent state of the disease, and lower values with prior history of splenectomy. The association with platelet count, an indirect marker of inflammatory activity in PV, supports the biological relevance of the ssGSEA signature; however, other validated inflammatory surrogates in PV—such as Absolute Neutrophil Count (ANC), reflecting JAK2-driven innate immune activation, and the Neutrophil-to-Lymphocyte Ratio (NLR), which captures systemic inflammatory and immune imbalance and is strongly associated with thrombotic risk and survival—were not available in the public clinical annotations of the analyzed datasets and therefore could not be assessed, underscoring the value of future studies integrating transcriptomic data with detailed inflammatory indices.

**Figure 6:**
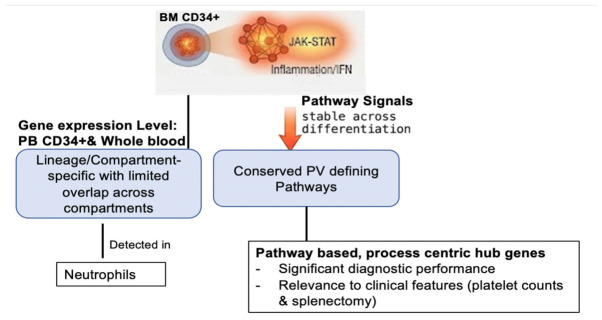
Summary of key transcriptomic findings across hematopoietic compartments in PV. The orange network represents disease-driving JAK–STAT, inflammatory, and IFN signaling in BM CD34+ progenitors. The downward arrow indicates propagation of these signaling programs into conserved pathways across differentiation, and the boxes represent downstream biological and clinical features.

Figure 6 presents a general scheme summarizing the biological framework emerging from this study. PV is initiated at the level of CD34+ progenitors by JAK–STAT-driven inflammatory programs that remain stable across hematopoietic differentiation. While the transcriptional output of these programs undergoes lineage- and compartment-specific modulation—resulting in limited overlap of individual differentially expressed genes—the underlying disease-defining pathways are conserved. These persistent inflammatory programs propagate into mature myeloid cells, enabling their detection in peripheral blood and neutrophils and supporting the development of pathway-based, process-centric biomarkers.

## 4. Materials and Methods

### 4.1. Integrated Cohort Composition

**Figure 7:**
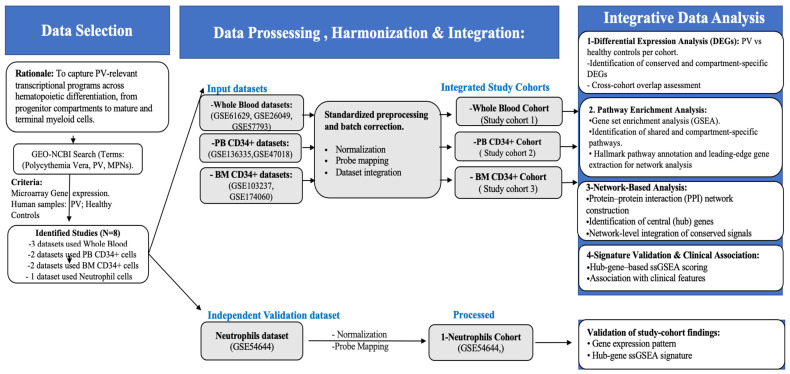
Schematic description of the study design.

To define the transcriptional landscape of PV, multiple independent GEO datasets for hematopoietic compartments, which compared PV and HC, were integrated after batch correction. The selected compartments capture transcriptional programs across key stages of myeloid differentiation in PV, including early progenitors (BM CD34+), circulating progenitors (PB CD34+), mixed mature populations (whole blood), and terminally differentiated neutrophils, enabling assessment of both compartment-specific and persistent disease-associated signatures. Whole blood merged from GSE61629 [26], GSE26049 [34], and GSE57793 [35]; PB CD34+ cells merged from GSE136335 [17] and GSE47018 [12]; and BM CD34+ cells merged from GSE103237 [36] and GSE174060 [15]. An independent neutrophil cohort (GSE54644 [37]) served as a validation cohort (Figure 7; Table 2). All samples were profiled on Affymetrix (Santa Clara, CA, USA) microarray platforms (Table 2). The list of samples included in the microarray datasets used to generate both the study cohorts and the validation cohort is summarized in Supplementary Tables S1–S4.

### 4.2. Microarray Data Processing

Microarray data processing was performed on the GenePattern platform (v3.9.11; Broad Institute, Cambridge, MA, USA) [38], a freely available web-based genomic analysis platform (https://www.genepattern.org/, accessed on 5 February 2026) that provides computational pipelines for gene expression analysis. Raw .CEL files were imported from NCBI-GEO and normalized using the ExpressionFileCreator or AffySTExpressionFileCreator modules for ST arrays with Robust Multi-Array Average (RMA) and background correction. Probes were collapsed to gene symbols based on the maximum-expressing probe, and datasets were log_2_-transformed and merged using the Merge Files function in AltAnalyze (v2.1.4; Gladstone Institutes, San Francisco, CA, USA) [39]. Batch correction was applied using the ComBat module [40] in GenePattern, generating final expression matrices for the whole blood, PB CD34+, and BM CD34+ cohorts. Differentially expressed genes (DEGs) were identified using ComparativeMarkerSelection. Venn diagrams were generated using InteractiVenn web tool (v1.0; interactivenn.net, accessed on 15 February 2026) to evaluate gene overlap across cohorts [41]. Volcano plots were created in the VolcaNoseR web tool (v1.0.3, https://huygens.science.uva.nl/VolcaNoseR/, accessed on 16 February 2026) [42]. Heatmaps were produced using the Heat Map Image module in GenePattern.

### 4.3. Gene Set Enrichment Analysis (GSEA)

The batch-corrected gene expression data in each study cohort were used to generate a ranked gene list within GSEA module in GenePattern. GSEA was performed using the Hallmark gene sets (h.all.v2025.1.Hs.symbols.gmt) [43] via the GSEA Preranked module. Subsequently, single-sample GSEA (ssGSEA) was conducted using a customized hub-gene list as the input gene-set database to compute ssGSEA enrichment scores. SRplot (v1.0; Tangruixuan Studio, Shanghai, China; http://www.bioinformatics.com.cn/SRplot, accessed on 19 February 2026) [44] was used to visualize ssGSEA score differences between PV and HCs using boxplots and to generate the receiver operating characteristic (ROC) curves. The area under the curve (AUC) was calculated to quantify the ability of genes and ssGSEA signatures to distinguish PV from HCs.

### 4.4. Protein–Protein Interaction (PPI) Analysis

A protein–protein interaction (PPI) network was constructed to analyze the functional relationships among the significantly enriched Hallmark gene sets. The network was generated using the Search Tool for the Retrieval of Interacting Genes/Proteins database (STRING) database (v12.0) via NetworkAnalyst web tool (v3.0; McGill University, Montreal, QC, Canada; networkanalyst.ca, accessed on 20 February 2026) [45], based on the leading-edge genes derived from the Hallmark gene sets commonly upregulated across the study cohorts. The analysis was limited to high-confidence, experimentally validated interactions (STRING interaction score ≥ 0.900). Nodes with a degree ≥ 10 were designated as hub genes and were selected for subsequent downstream analysis.

### 4.5. Statistical Analysis

Statistical analyses for assessing differences in gene expression and gene set enrichment were performed using GenePattern v3.9.11 (Broad Institute, Cambridge, MA, USA). A two-sided *t*-test implemented in the ComparativeMarkerSelection module was used to evaluate differential gene expression. Thresholds of log_2_ fold change > ±1 and –log_10_(*p*-value) > 2 were applied to define statistical significance. For GSEA, gene sets with a false discovery rate (FDR) < 0.25 were considered significantly enriched, and normalized enrichment scores (NESs) were used to indicate the direction and magnitude of enrichment. The Mann–Whitney U test was applied to compare ssGSEA scores across clinical categories, while associations between continuous clinical parameters and ssGSEA scores were evaluated using Spearman’s rank correlation coefficient (Rho) in Statisty, a web tool available at (https://statisty.app/, accessed on 21 February 2026). A *p*-value < 0.05 was considered statistically significant for comparisons of ssGSEA scores between PV and HCs, as well as for analyses of categorical and continuous clinical feature.

## 5. Conclusions

In summary, this work supports a model in which PV is driven by a stable, progenitor-derived inflammatory program that persists through differentiation while incorporating compartment-specific adaptations. By integrating transcriptomic data from progenitor and mature myeloid compartments, this study defines a conserved network-based inflammatory signature with consistent diagnostic performance across compartments, supporting its relevance to core disease biology. Limitations include dataset heterogeneity, bulk transcriptomic design, and lack of single-cell resolution. Nevertheless, the consistency across compartments supports the value of multi-compartment, network-based transcriptomic approaches for improving biological understanding and biomarker discovery in PV and provides a foundation for future mechanistic and translational studies.

## Figures and Tables

**Table 1:** The relationship between ssGSEA score of hub genes and clinical features.

Clinical Feature	ssGSEA Score: Median (IQR)	Mann–Whitney U Test	*p*-Value
Female (*n* = 11) vs. Male (*n* = 8)	Female: 2924.6 (1024) Male: 2843.4 (642.43)	43	0.934
Splenectomy (No: *n* = 14/Yes: *n* = 5)	No: 2948.8 (851.04) Yes: 2460 (516.48)	13	**0.042**
Thrombosis (No: *n* = 14/Yes: *n* = 5)	No: 2948.8 (833.1) Yes: 2594.8 (320.4)	17	0.096
Acute Leukemia (No: *n* = 14/Yes: *n* = 5)	No: 2948.8 (798.46) Yes: 2780.48 [524.34]	20	0.165
Aggressiveness (Indolent: *n* = 12/Aggressive: *n* = 7)	Indolent: 3191.7 (823.2) Aggressive: 2594.8 (422.4)	13	**0.014**
**Variable**	**Median (IQR) [min–max]**	**Rho (Spearman Correlation)**	***p*-value**
ssGSEA Score	2898.4 (837) [1662.9–4383.4]	-	-
Age (years)	66 (15.5) [46–82]	−0.03	0.915
JAK2 V617F burden (%)	100 (32.5) [55–100]	−0.32	0.174
Disease Duration (years)	9 (10) [1–25]	−0.06	0.805
Hemoglobin (g/dL)	12.5 (2.55) [8.3–15.9]	0.26	0.284
Leukocyte Count (×10^3^/uL)	17.6 (8730) [4430–177.1]	−0.25	0.293
Platelet Count (×10^3^/uL)	712 (633.5) [151–1480]	0.46	**0.049**

Bold indicates statistically significant *p*-values (*p* < 0.05) and section headers distinguishing the upper (group comparisons) and lower (correlation analysis) sections. IQR—interquartile range; min—minimum; max—maximum.

**Table 2:** Summary of GEO microarray datasets included in the analysis.

Dataset		PV	Controls	Platforms	Hematopoietic Compartment/Biological Rationale
Study Cohort 1	**1-Whole Blood Datasets**				
	GSE61629	21	21	Affymetrix Human Genome U133 Plus 2.0 Array	Mixed mature circulating cells; system-level disease manifestation
	GSE26049	41	21	Affymetrix Human Genome U133 Plus 2.0 Array	
	GSE57793	21	-	Affymetrix Human Genome U133 Plus 2.0 Array	
Study Cohort 2	**2-PB CD34+ Datasets**				
	GSE136335	3	6	Affymetrix Human Transcriptome Array 2.0 [HTA-2.0]	Circulating hematopoietic progenitors; clonal propagation
	GSE47018	19	7	Affymetrix Human Genome U133A Array	
Study Cohort 3	**3-BM CD34+ Datasets**				
	GSE103237	26	15	Affymetrix Human Genome U219 Array	Bone-Marrow progenitors; disease origin
	GSE174060	8	-	Affymetrix Human Transcriptome Array 2.0 [HTA-2.0]	
Validation Cohort	**4-Neutrophils Dataset**				
	GSE54644	28	11	Affymetrix GeneChip HT-HG_U133A Array	Terminal myeloid effector cells; Validation

Bold indicates hematopoietic compartment cohorts included in the study.

## Data Availability

The original contributions presented in this study are included in the article. Further inquiries can be directed to the corresponding author.

## Supplementary Materials

The following supporting information can be downloaded at: https://www.mdpi.com/article/10.3390/ijms27104580/s1.

## Abbreviations

AUC	Area under the (ROC) curve
BM	Bone marrow
CD34+	Cluster of differentiation 34–positive
CEL	Affymetrix raw array file format (.CEL)
ComBat	Combating Batch Effects
DEG/s	Differentially expressed gene(s)
E2F	E2F transcription factor family
FC/log_2_FC	Fold change/log_2_ fold change
FDR	False discovery rate
GEO	Gene Expression Omnibus
GSEA	Gene Set Enrichment Analysis
G2/M	G2/M cell-cycle checkpoint
HC	Healthy controls
IFN-α/IFN-γ	Interferon-alpha/Interferon-gamma
IL-6	Interleukin-6
IQR	Interquartile range
JAK–STAT	Janus kinase–signal transducer and activator of transcription
JAK2V617F	JAK2 c.1849G>T (Val617Phe) mutation
MYC	MYC proto-oncogene transcriptional program
MPN	Myeloproliferative Neoplasm
NES	Normalized enrichment score
NETosis	Neutrophil extracellular trap formation
NF-κB	Nuclear factor kappa-light-chain-enhancer of activated B cells
PB	Peripheral blood
PB CD34+	Peripheral-blood CD34+ cells
PPI	Protein–protein interaction
PV	Polycythemia vera
RMA	Robust Multi-Array Average
ROC	Receiver operating characteristic
ssGSEA	Single-sample Gene Set Enrichment Analysis
STAT	Signal transducer and activator of transcription
STRING	Search Tool for the Retrieval of Interacting Genes/Proteins database
*t*-test	Two-sided Student’s *t*-test
VolcaNoseR	Web tool for volcano plot visualization
–log_10_(*p*)	Negative base-10 logarithm of *p*-value
ρ (rho)	Spearman’s rank correlation coefficient
