# Supplementary material for: Multi-Compartment Transcriptomics Identifies a Persistent Inflammatory Program and a Network-Derived Diagnostic Signature in Polycythemia Vera
Source: Int J Mol Sci. 2026 May 20;27(10):4580. doi: 10.3390/ijms27104580 (PMC13207336; doi:10.3390/ijms27104580)
Supplement: Supplementary file 1 [file ijms-27-04580-s001.zip › ijms-4223089-supplementary.pdf]

Supplementary Table S1. Microarray Gene Expression Datasets Used to Generate the Whole-Blood Cohort

	Whole Blood Datasets					
#	GSE61629		GSE26049		GSE57793	
1	PV_202_D1	GSM1388590	RMA PV_1	GSM639630	PV_250_D2	GSM1388597
2	PV_228_K1	GSM1388591	RMA PV_2	GSM639631	PV_275_K2	GSM1388602
3	PV_230_B1	GSM1388592	RMA PV_3	GSM639632	PV_314_O2	GSM1388609
4	PV_233_L1	GSM1388593	RMA PV_4	GSM639633	PV_316_B2	GSM1388610
5	PV_234_M1	GSM1388594	RMA PV_5	GSM639634	PV_319_P2	GSM1388611
6	PV_238_O1	GSM1388595	RMA PV_6	GSM639635	PV_328_F2	GSM1388612
7	PV_242_P1	GSM1388596	RMA PV_7	GSM639636	PV_330_R2	GSM1388613
8	PV_258_F1	GSM1388598	RMA PV_8	GSM639637	PV_338_L2	GSM1388615
9	PV_260_Q1	GSM1388599	RMA PV_9	GSM639638	PV_356_Q2	GSM1388617
10	PV_262_R1	GSM1388600	RMA PV_10	GSM639639	PV_357_U2	GSM1388618
11	PV_269_S1	GSM1388601	RMA PV_11	GSM639640	PV_373_M2	GSM1388619
12	PV_280_Z1	GSM1388603	RMA PV_12	GSM639641	PV_385_S2	GSM1388620
13	PV_283_U1	GSM1388604	RMA PV_13	GSM639642	PV_405_Z2	GSM1388621
14	PV_285_T1	GSM1388605	RMA PV_14	GSM639643	PV_417_X2	GSM1388622
15	PV_302_X1	GSM1388606	RMA PV_15	GSM639644	PV_467_T2	GSM1388625
16	PV_303_I1	GSM1388607	RMA PV_16	GSM639645	PV_482_I2	GSM1388626
17	PV_304_Y1	GSM1388608	RMA PV_17	GSM639646	PV_490_AD2	GSM1388627
18	PV_332_AC1	GSM1388614	RMA PV_18	GSM639647	PV_497_Y2	GSM1388628
19	PV_348_AD1	GSM1388616	RMA PV_19	GSM639648	PV_504_AG2	GSM1388629
20	PV_434_AG1	GSM1388623	RMA PV_20	GSM639649	PV_507_AC2	GSM1388630
21	PV_446_AH1	GSM1388624	RMA PV_21	GSM639650	PV_510_AH2	GSM1388631
22	Control_1	GSM1509517	RMA PV_22	GSM639651		
23	Control_2	GSM1509518	RMA PV_23	GSM639652		
24	Control_3	GSM1509519	RMA PV_24	GSM639653		
25	Control_4	GSM1509520	RMA PV_25	GSM639654		
26	Control_5	GSM1509521	RMA PV_26	GSM639655		
27	Control_6	GSM1509522	RMA PV_27	GSM639656		
28	Control_7	GSM1509523	RMA PV_28	GSM639657		
29	Control_8	GSM1509524	RMA PV_29	GSM639658		
30	Control_9	GSM1509525	RMA PV_30	GSM639659		
31	Control_10	GSM1509526	RMA PV_31	GSM639660		
32	Control_11	GSM1509527	RMA PV_32	GSM639661		
33	Control_12	GSM1509528	RMA PV_33	GSM639662		
34	Control_13	GSM1509529	RMA PV_34	GSM639663		
35	Control_14	GSM1509530	RMA PV_35	GSM639664		
36	Control_15	GSM1509531	RMA PV_36	GSM639665		
37	Control_16	GSM1509532	RMA PV_37	GSM639666		
38	Control_17	GSM1509533	RMA PV_38	GSM639667		
39	Control_18	GSM1509534	RMA PV_39	GSM639668		
40	Control_19	GSM1509535	RMA PV_40	GSM639669		
41	Control_20	GSM1509536	RMA PV_41	GSM639670		
42	Control_21	GSM1509537	RMA Control_1	GSM639681		
43			RMA Control_2	GSM639682		
44			RMA Control_3	GSM639683		
45			RMA Control_4	GSM639684		
46			RMA Control_5	GSM639685		
47			RMA Control_6	GSM639686		
48			RMA Control_7	GSM639687		
49			RMA Control_8	GSM639688		
50			RMA Control_9	GSM639689		
51			RMA Control_10	GSM639690		
52			RMA Control_11	GSM639691		
53			RMA Control_12	GSM639692		
54			RMA Control_13	GSM639693		
55			RMA Control_14	GSM639694		
56			RMA Control_15	GSM639695		
57			RMA Control_16	GSM639696		
58			RMA Control_17	GSM639697		
59			RMA Control_18	GSM639698		
60			RMA Control_19	GSM639699		
61			RMA Control_20	GSM639700		
62			RMA Control_21	GSM639701		

Supplementary Table S2. Microarray Gene Expression Datasets Used to Generate the PB CD34+ Cohort

	PB CD34+ Datasets			
#	GSE136335		GSE47018	
1	PV1	GSM4046643	UPIN 1020	GSM1143005
2	PV9	GSM4046653	UPIN 1045	GSM1143006
3	PV11	GSM4046657	UPIN 121	GSM1143007
4	HC4	GSM4046662	UPIN 132	GSM1143008
5	HC5	GSM4046664	UPIN 136	GSM1143009
6	HC6	GSM4046667	UPIN 173	GSM1143010
7	HC7	GSM4046668	UPIN 183-1a	GSM1143011
8	HC8	GSM4046670	UPIN 190	GSM1143013
9	HC9	GSM4046672	UPIN 206	GSM1143014
10			UPIN 223	GSM1143015
11			UPIN 234	GSM1143016
12			UPIN 253	GSM1143017
13			UPIN 564	GSM1143018
14			UPIN 592	GSM1143019
15			UPIN 684-1a	GSM1143020
16			UPIN 684-2a	GSM1143021
17			UPIN 890	GSM1143022
18			UPIN 905	GSM1143023
19			UPIN 906	GSM1143024
20			UPIN 929	GSM1143025
21			UPIN 931	GSM1143026
22			UPIN 950	GSM1143027
23			UPIN 957	GSM1143028
24			UPIN 983	GSM1143029
25			UPIN 993	GSM1143030
26			UPIN 997	GSM1143031

Supplementary Table S3. Microarray Gene Expression Datasets Used to Generate the BM CD34+ Cohort

	PM CD34+ Datasets			
#	GSE103237		GSE174060	
1	patient 4763	GSM2758703	PV2	GSM5285418
2	patient 4764	GSM2758704	PV3	GSM5285419
3	patient 4765	GSM2758705	PV4	GSM5285420
4	patient 5772	GSM2758706	PV5	GSM5285421
5	patient 5811	GSM2758707	PV7	GSM5285422
6	patient 5827	GSM2758708	PV8	GSM5285423
7	patient 5883	GSM2758709	PV10	GSM5285425
8	patient 5884	GSM2758710	PV12	GSM5285427
9	patient 5903	GSM2758711		-
10	patient 5907	GSM2758712		-
11	patient 5933	GSM2758713		-
12	patient 5983	GSM2758714		_
13	patient 5995	GSM2758715		_
14	patient 7025	GSM2758716		_
15	patient 7028	GSM2758717		_
16	patient 7083	GSM2758718		_
17	patient 7124	GSM2758719		_
18	patient 7144	GSM2758720		_
19	patient 7153	GSM2758721		_
20	patient 7164	GSM2758722		_
21	patient 7201	GSM2758723		_
22	patient 10_591	GSM2758724		_
23	patient 11_122	GSM2758725		_
24	patient 11_202	GSM2758726		_
25	patient 11_46	GSM2758727		_
26	patient 5764	GSM2758728		_
27	Healthy Donor 1	GSM2758729		_
28	Healthy Donor 10	GSM2758730		
29	Healthy Donor 11	GSM2758731		
30	Healthy Donor 12	GSM2758732		
31	Healthy Donor 13	GSM2758733		
32	Healthy Donor 14	GSM2758734		
33	Healthy Donor 15	GSM2758735		
34	Healthy Donor 2	GSM2758736		
35	Healthy Donor 3	GSM2758737		
36	Healthy Donor 4	GSM2758738		
37	Healthy Donor 5	GSM2758739		
38	Healthy Donor 6	GSM2758740		
39	Healthy Donor 7	GSM2758741		
40	Healthy Donor 8	GSM2758742		
41	Healthy Donor 9	GSM2758743		

Supplementary Table S4. Microarray Gene Expression Dataset Used to Generate the Neutrophil Validation Cohort

#	Neutrophils Validation Cohort	
1	GSM1320525	diagnosis: PV
2	GSM1320529	diagnosis: PV
3	GSM1320530	diagnosis: PV
4	GSM1320533	diagnosis: PV
5	GSM1320534	diagnosis: PV
6	GSM1320541	diagnosis: PV
7	GSM1320544	diagnosis: PV
8	GSM1320546	diagnosis: PV
9	GSM1320549	diagnosis: PV
10	GSM1320550	diagnosis: PV
11	GSM1320555	diagnosis: PV
12	GSM1320556	diagnosis: PV
13	GSM1320560	diagnosis: PV
14	GSM1320561	diagnosis: PV
15	GSM1320562	diagnosis: PV
16	GSM1320564	diagnosis: PV
17	GSM1320565	diagnosis: PV
18	GSM1320568	diagnosis: PV
19	GSM1320569	diagnosis: PV
20	GSM1320571	diagnosis: PV
21	GSM1320573	diagnosis: PV
22	GSM1320574	diagnosis: PV
23	GSM1320575	diagnosis: PV
24	GSM1320577	diagnosis: PV
25	GSM1320578	diagnosis: PV
26	GSM1320584	diagnosis: PV
27	GSM1320592	diagnosis: PV
28	GSM1320593	diagnosis: PV
29	GSM1320617	diagnosis: Normal
30	GSM1320618	diagnosis: Normal
31	GSM1320619	diagnosis: Normal
32	GSM1320620	diagnosis: Normal
33	GSM1320621	diagnosis: Normal
34	GSM1320622	diagnosis: Normal
35	GSM1320623	diagnosis: Normal
36	GSM1320624	diagnosis: Normal
37	GSM1320625	diagnosis: Normal
38	GSM1320626	diagnosis: Normal
39	GSM1320627	diagnosis: Normal
